# Inner Profile Measurement for Pipes Using Penetration Testing

**DOI:** 10.3390/s19020237

**Published:** 2019-01-10

**Authors:** Robert Ross, Avinash Baji, Dean Barnett

**Affiliations:** 1 Department of Engineering, La Trobe University, 3086 Melbourne, Australia; A.Baji@latrobe.edu.au; 2 Western Water and Intelligent Water Networks, 3429 Melbourne, Australia; Dean.Barnett@westernwater.com.au

**Keywords:** concrete inspection, condition assessment, penetration testing, concrete probing, remote sensing

## Abstract

Penetration testing has been used to measure material properties for over fifty years. Currently, it is under-utilised as a contemporary scientific and engineering tool for investigating the condition of pipes whose inner surface has been exposed to chemical attack. We describe the design, development and calibration of a portable probe which uses a penetrative strain gauge load cell to measure where the semi-solid surface starts and stops within a pipe. We also describe the results of field tests of the probe in concrete sewers, affected by internal corrosion, where the probe proved to be a fast and reliable method for collecting pipe profile information. The results indicate significant benefit in the use of penetrometers to perform concrete sewer condition assessment.

## 1. Introduction

Penetration testing for characterising materials is a proven technology in a variety of contexts, including soils [1], frozen geomaterials [2], insect studies [3], even spacecraft landing [4]. For pipes that maintain their physical state as a pristine mathematical cylinder, with constant diameter, there would be little reason to measure them using penetration testing. However, pipes used in operational systems are often in a somewhat-less-than-pristine state, particularly when their purpose is to transport a mix of chemicals that ultimately limit the service life of these pipes via chemical attack.

To define chemical attack, we take the engineering point of view that the interaction between the chemicals and the pipe must be destructive, in that the result of this interaction must have resulted in pipe deterioration and/or loss of durability [5]. For example, the consistency of the corroding inner surface of concrete sewer pipes has often been likened to a soft cottage cheese (e.g., [6]). Furthermore, over its lifetime, a pipe may have potentially experienced varying types of attacks to different depths and therefore may exhibit layers of damage [7].

Non-contact testing is a valuable tool for inspecting the internal profile of a pipe, but the information it provides is not necessarily complete. In the context of sewers, a variety of tests have been operationalised (e.g., [8]) to augment traditional CCTV. Looks may be deceiving to some degree—a surface that appears to be hard may be soft, and a surface that appears to be soft might not be under chemical attack. Simply measuring the optical profile of pipes (e.g., [9,10]) might not provide sufficient information to correctly prioritise pipe rehabilitation works.

This paper is structured as follows. In Section 2, we discuss the requirements for measuring the profile of the inner surface of pipes. In Section 3, we discuss the design of a surface-contacting penetrometer to meet these requirements. In Section 4, we discuss bench-scale and pilot tests of our system. Finally, we reflect on the degree to which our requirements were met and discuss future directions in Section 5.

## 2. Requirements

**Figure 1:**
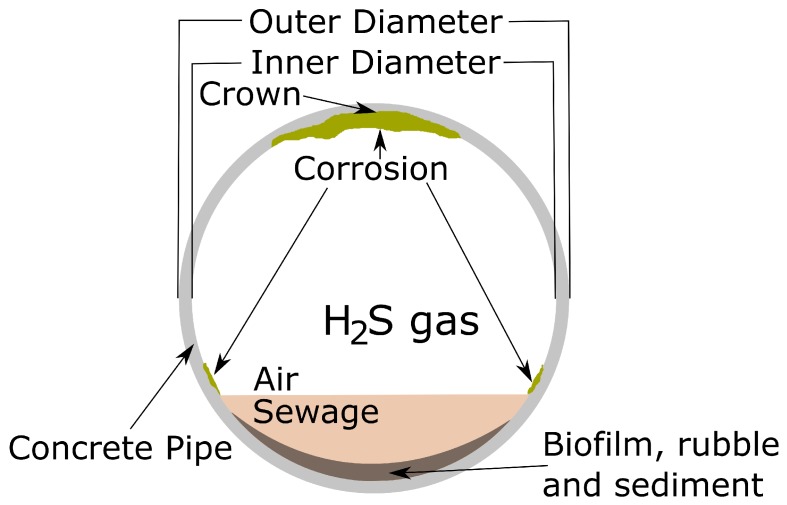
Diagram of the corroded sewer pipe context.

Following in the footsteps of others (e.g., [11]), our penetrometer is designed to fulfil three requirements in terms of size, rigidity and velocity. With size, the measuring tip was devised to be of a similar or smaller vertical dimension than the layers it needs to detect; having a small diameter and length dimension to allow detection of thin layers and the breaking of grain bonds rather than producing a primarily compressive failure. This advice was given in the context of performing high spatial resolution stratigraphy of snow. However, our application context of sewer pipes is substantially different to snow, with the harsh domain (see Figure 1) requiring a significantly more ruggedised tip, so rigidity was prioritised over size [6]. Note the generous amount of corrosion depicted at the top (obvert) of the crown region of the pipe. Consequently, we firstly built a penetrometer that was fit for purpose within this environment, then assessed whether it can detect layers.

**Figure 2:**
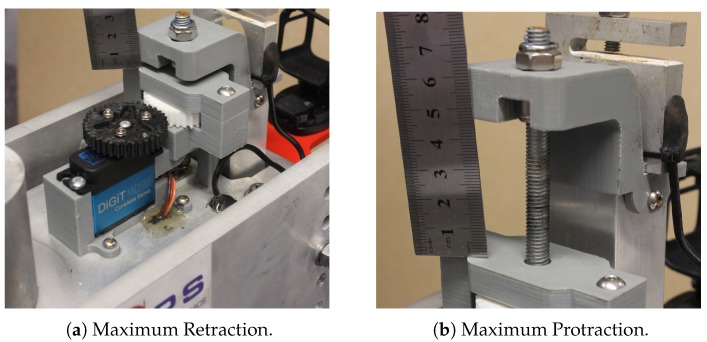
Photographs of penetrometer extremes.

In terms of the size constraints of our application context, we intended for our penetrometer to be able to work in concrete sewer pipes, with the smallest target internal pipe diameter being 225 mm with an associated external pipe diameter of 295 mm. As an aside, these pipes can corrode reasonably quickly, various studies have discovered that up to 4.7 mm/year is possible (e.g., [12]). Consequently, we designed our probe to protract up to 40 mm (see Figure 2). With a normal pipe width of 70 mm and a protraction distance of 40 mm, our penetrometer will not reach the outer wall of a pipe, unless it is also corroded on the outside. Our penetrometer, therefore, will be generally non-destructive when operated in minimum-width concrete pipes.

With the latter two advised requirements of rigidity and velocity, we agree that high rigidity and constant velocity penetration minimise measurement inaccuracies that arise from the storage and release of energy during penetration and inertial forces caused by sudden penetration velocity changes. Constant-velocity penetration (1 mm per second) avoids the rate-dependence associated with creep deformation, assuming a homogeneous material, which is not always the case in concrete sewer pipes.

## 3. Design

**Figure 3:**
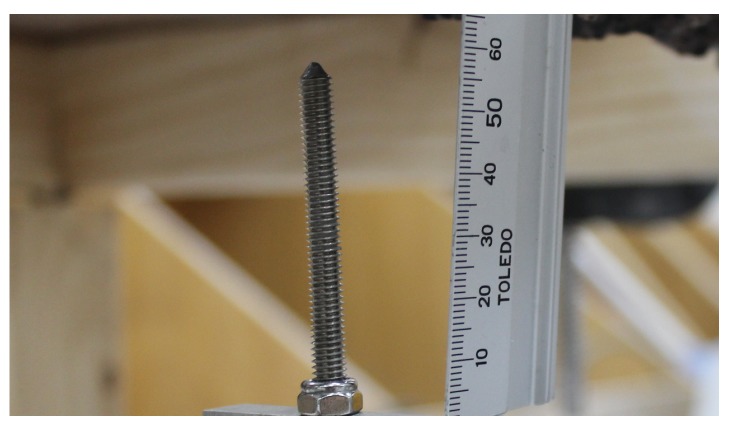
The penetrometer tip.

We now describe the materials, sensing and actuator components used by the penetrator. Our design was strongly influenced by the harsh requirements of the proposed operating environment, and less influenced by design standards for penetrometers in other domains (e.g., [13]). To transmit the force exerted by the surface material to the sensing element in a reliable fashion, our probe has a highly rigid tip (see Figure 3) a 45${}^{\circ }$ angle milled into the top of a stainless steel screw. Aluminium, while lighter, was judged to be of insufficient strength when repeatedly forced up against a concrete sewer pipe, hence the choice of a stainless steel tip to provide strength and corrosion resistance. This design was chosen for two reasons: it is safer during manual handling if the head is not exceedingly sharp; and it has some capacity to cope with a angled surface and align the force applied by the probe to the concrete surface with the centre of the shaft, which is required to obtain a correct reading from the load cell.

**Figure 4:**
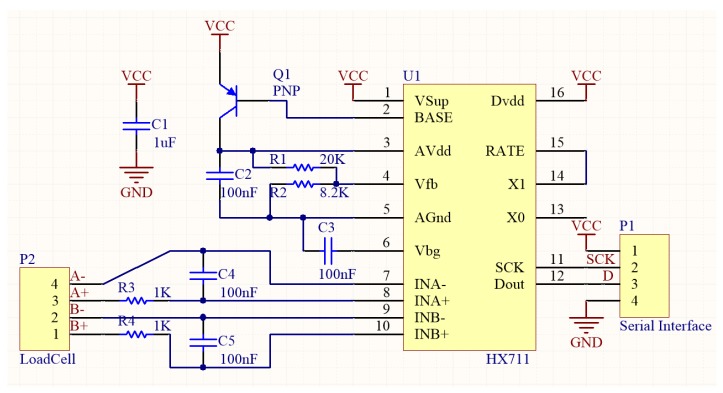
Circuit diagram for the strain gauge load cell, showing the Wheatstone Bridge.

The penetrometer measures force using four strain gauges in a Wheatstone bridge configuration within a S-shaped block of material, an off-the-shelf Shenlan LCS 550 10 kg load cell (P2), which has a resolution of ±5 g. The change in resistance of the strain gauge provides an electrical value change proportional to the load placed on the cell which is then amplified (the HX711) and scaled into a usable range (see Figure 4). Given the harshness of the application context, the primary purpose of the load cell was to detect when the probe had made contact with the solid surface of the pipe because (unlike snow) protraction much further could destroy the penetrometer.

**Figure 5:**
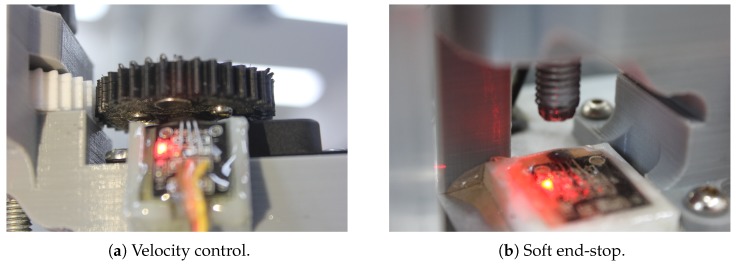
Photos of the Hall-effect sensors.

The penetrometer measures distance using an off-the-shelf Hall-effect sensor (A3144E) which detects three magnets in close proximity on the underside of the drive gear which provides the constant velocity (see Figure 5), all within an epoxy protective casing. Our configuration provided a penetration depth measurement resolution of 0.42mm (based on the pitch of the screw thread and encoder resolution). Once the maximum distance is reached, the penetrometer automatically retracts back to its starting position. To control this position, we used another set of Hall-effect sensors as a soft end stop.

**Figure 6:**
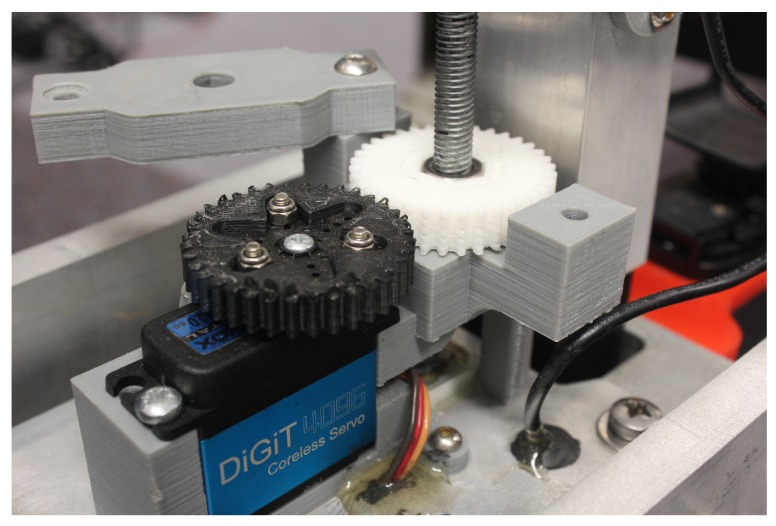
Photo of the protraction actuator.

The protraction actuator is driven by a lead-screw nut which is coupled to a waterproof servo motor [14] via a pair of 3D-printed nylon spur-gears (see Figure 6). The probe slides up and down in a single axis, as required by the strain gauge, within a prismatic joint. The materials selected (an aluminium slider on a Delrin rod), require little lubrication and are suitable for the harsh environment.

**Figure 7:**
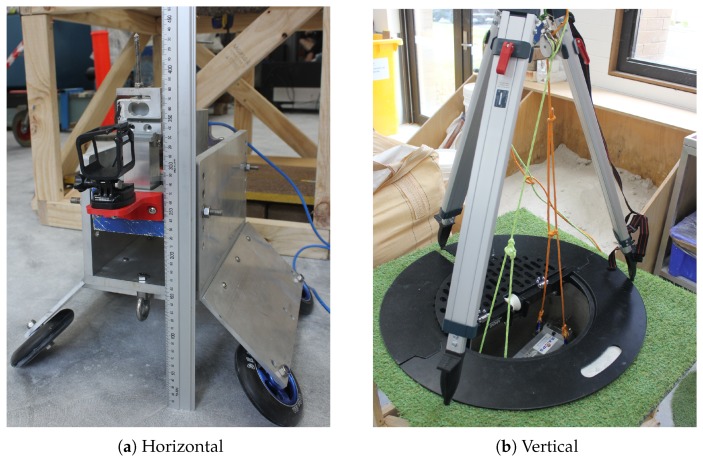
Penetrometer mounts for different pipe orientations.

Designed to operate within the two main dimensions of concrete sewers, our penetrometers were mounted onto two different rigs: one for measuring the surface profile in vertical concrete sewer pipes, and one for measuring the surface profile in horizontal concrete sewer pipes. The vertical rig consisted of a long extendible block (see Figure 7) which was designed to be lowered into and penetrate the side wall of a sewer access point. The horizontal rig consisted of our penetrometer mounted on a solid trolley with skateboard wheels (see Figure 7) and pulled between two sewer access points using ropes (floated between points prior to penetration testing) or tethered to another robot. Different sized wheels and mounts could be used to position the probe near the inner surface, depending on the size of the pipe.

## 4. Results

**Figure 8:**
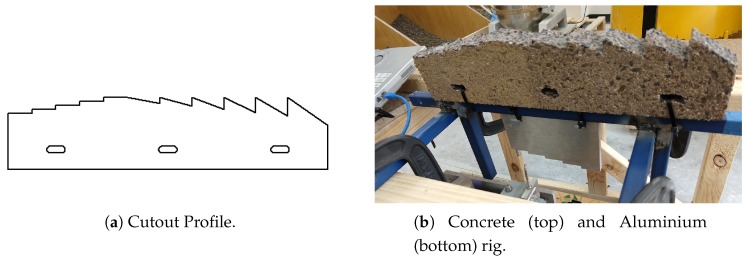
Cutouts for benchmarking the penetrometer.

We firstly bench tested our penetrometer with respect to its ability to measure height, force, and materials, before taking our penetrometers into a concrete sewer system and trialling them against horizontal sections of pipes. In order to benchmark our penetrometer against height, we waterjet cut out a piece of aluminium (as our ground truth) in four step increments of 5 mm, using the left half of the SolidWorks profile shown in Figure 8.

**Figure 9:**
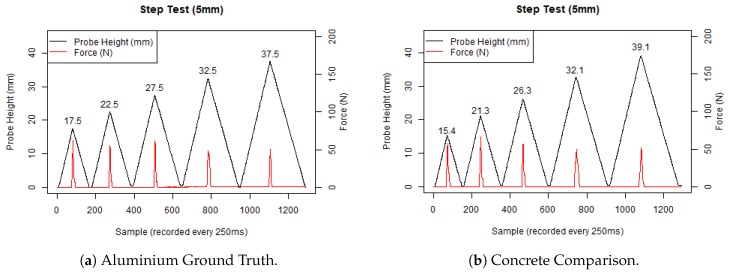
Step benchmarking tests.

It is evident from Figure 9a that our penetrometer recorded measurements of exactly 5 mm between the different smooth aluminium step levels, from 17.5 mm to 37.5 mm. We also ran the same test against a concrete paver waterjet cut using the same template. The cut out had a much bumpier surface, shown in the data of Figure 9b; as expected, the surface has a similar trend.

The concrete paver is longer than the aluminium ground truth because we also cut six different test angles into the concrete (from 10–35 degrees). Concrete sewers generally do not have perfectly horizontal surfaces, so we wanted to determine whether there were any changes in velocity for our penetrometer that might impact our measurements, but more importantly, if our device could withstand slipping around off the rough surface of a concrete sewerage pipe.

**Figure 10:**
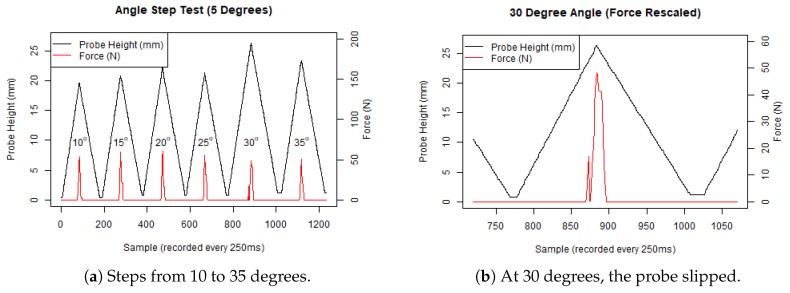
Angled concrete benchmarking tests.

The penetrometer handled the rough angled concrete surfaces reasonably well, as shown in Figure 10a,b. The penetrometer slipped the most on the 30 degree test, on a small piece of aggregate, evidenced by the small spike in the force measured (Figure 10b) and also seen in the greater probe height recorded (Figure 10a).

**Figure 11:**
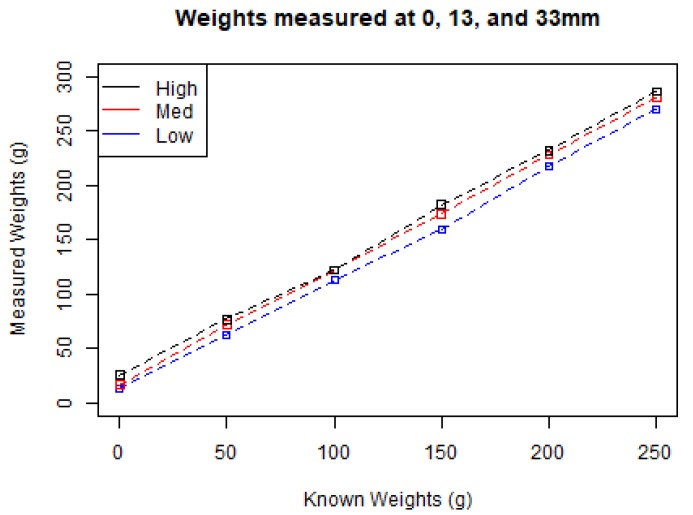
Difference between measured and known weights (g) at three protraction heights.

We next proceeded to investigate our penetrometer’s ability to measure force. We applied five weights, from 50 to 250 g, across three levels of protraction height (Low = 0 mm, Med = 13 mm, High = 33 mm) and record the results in Figure 11. These results demonstrate a linear relationship between weight applied and force measured (within the accuracy range of the penetrometer) which is independent of probe height.

**Figure 12:**
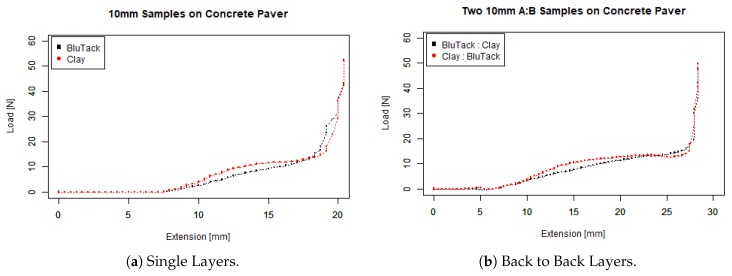
Penetrometer testing soft layers on concrete.

**Figure 13:**
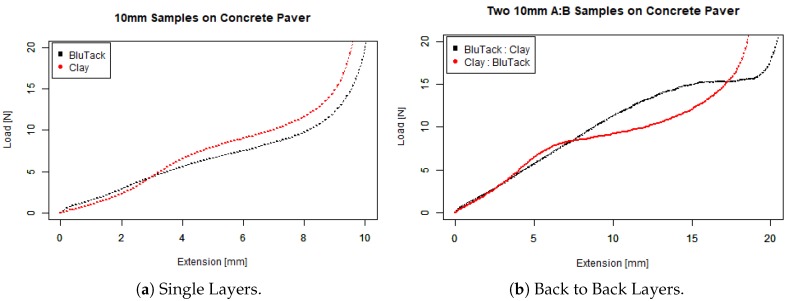
Instron 5980 testing soft layers on concrete.

To determine the penetrometer’s ability to distinguish between materials, we recorded the amount of force required to indent materials that we were advised were similar to corroded concrete, BluTack and modelling clay. We also wanted to determine the degree to which our penetrometer could profile corroded surfaces with different layers, so we constructed samples with BluTack and modelling clay in different orders. We benchmarked our penetrometer (see Figure 12) against the Instron 5980 Test machine (see Figure 13) with a similar penetration velocity of 1 mm per second.

As expected, and evident from the slope of load versus displacement curves, (a) shows that the Instron more easily penetrates 10 mm BluTack (lower black line) as opposed to 10 mm modelling clay (higher red line). For example, at a depth of 6 mm the load recorded for BluTack is about 7.5 N and at a similar penetration depth for clay the load value is closer to 8.5 N. With layers (b), it is difficult to identify a smooth transition from BluTack to Clay (Black Line), we believe that these materials are behaving as a single composite, potentially due to the confinement effect. On the other hand, there is a clear transition from Clay to BluTack (Red Line) where the slope flattens after 10 mm of penetration depth. Consequently, we believe that our penetrometer can detect layering, with early flattening indicating a transition from harder to softer materials and vice versa. Note that for both tests we set the Instron to push all the way through to concrete, which the Instron identified at 20 N when the curve goes towards vertical. To be completely sure, we set the stopping criteria of the penetrometer at 50 N, as seen in the previous graphs on step and angle tests.

There were similarities between the data collected by the penetrometer and the Instron. The load versus displacement curves by the penetrometer were very similar to the Instron curves, and the trend was also similar between the layered data. There are some differences in the graphs, due to differences in the experimental set-up between the Instron and the penetrometer. The initial position of the Instron probe was extremely close to the surface of the test samples, pointing down, whereas the position of the penetrometer was about 7.5 mm away from the test samples, pointing up. The effect on the data is that penetration takes some time to occur, thus the curves are scaled lower.

**Figure 14:**
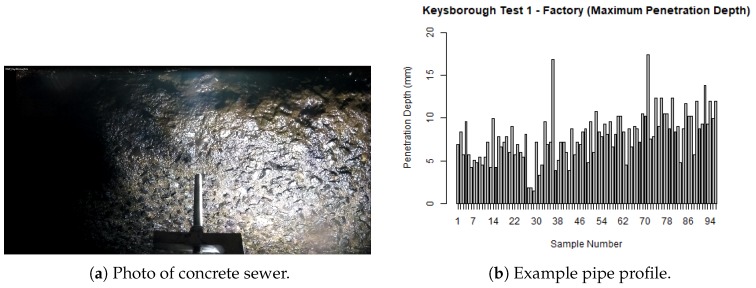
Profiling horizontal concrete sewer pipes.

Having characterised our penetrometer in the laboratory, we then took our devices into a concrete sewer in conjunction with operational personnel using standard access procedures. We operated our devices on multiple horizontal runs with pipe diameters from 225 mm through to 525 mm, on 100 m sections of sewer in the suburbs of Bacchus Marsh and Keysborough. Samples were taken at 1 m, an example of the Keysborough run is shown in Figure 14.

This run is interesting to sewer maintenance practitioners for several reasons. There are two points where corrosion exceeds 15 mm, and there is a fair amount of corrosion over 5 mm. Also, the maximum amount of time required to take an individual sample was 30 s.

These pipes were over 50 years old and had exposed aggregate, visually suggesting that some concrete corrosion has occurred. Experimentally, we have observed that degradation at times can be localised down to small segments of pipe, hence we would expect a probing regime to operate on a regular basis (e.g., probe conducted every meter) which will support the use of analytical models of the service life of systems of pipes.

## 5. Conclusions

A penetrometer for profiling the inner surface of pipes has been designed and two example systems have been road tested in the context of corroded concrete sewer pipes. We expect that penetrometer-based condition assessment, coupled with traditional visual inspection, will find practical applicability in a range of different concrete pipe environments.

We have identified significant scope for improving the serviceability and operation of the proposed system including ruggedisation, projected life-cycle testing, explosive environmental certification, facilitation of a larger range of pipe sizes and tilting of the sensor to allow different test points away from the object to be measured.
